# High-precision microbeam radiotherapy reveals testicular tissue-sparing effects for male fertility preservation

**DOI:** 10.1038/s41598-019-48772-3

**Published:** 2019-10-01

**Authors:** Hisanori Fukunaga, Kiichi Kaminaga, Takuya Sato, Karl T. Butterworth, Ritsuko Watanabe, Noriko Usami, Takehiko Ogawa, Akinari Yokoya, Kevin M. Prise

**Affiliations:** 1 Centre for Cancer Research and Cell Biology, Queen’s University Belfast, 97 Lisburn Road, Belfast, BT9 7AE UK; 2 Shonan Kamakura General Hospital, 1370-1 Okamoto, Kamakura, Kanagawa, 247-8533 Japan; 3 Institute for Quantum Life Science, National Institutes for Quantum and Radiological Science and Technology, 2-4 Shirakata-Shirane, Tokai, Ibaraki, 319-1195 Japan; 4 Institute of Molecular Medicine and Life Science, Yokohama City University Association of Medical Science, 3-9 Fukuura, Kanazawa-ku, Yokohama, 236-0004 Japan; 5 Institute of Materials Structure Science, High Energy Accelerator Research Organization, 1-1 Oho, Tsukuba, Ibaraki, 305-0801 Japan

**Keywords:** Cancer models, Self-renewal

## Abstract

Microbeam radiotherapy (MRT) is based on a spatial fractionation of synchrotron X-ray microbeams at the microscale level. Although the tissue-sparing effect (TSE) in response to non-uniform radiation fields was recognized more than one century ago, the TSE of MRT in the testes and its clinical importance for preventing male fertility remain to be determined. In this study, using the combination of MRT techniques and a unique *ex vivo* testes organ culture, we show, for the first time, the MRT-mediated TSE for the preservation of spermatogenesis. Furthermore, our high-precision microbeam analysis revealed that the survival and potential migration steps of the non-irradiated germ stem cells in the irradiated testes tissue would be needed for the effective TSE for spermatogenesis. Our findings indicated the distribution of dose irradiated in the testes at the microscale level is of clinical importance for delivering high doses of radiation to the tumor, while still preserving male fertility.

## Introduction

Infertility is still an unfortunate adverse effect of most cancer therapies, as it impacts the quality of life in cancer survivors. In recent clinical practice, radiation treatments for cancer have evolved to a high level of precision and accuracy, nevertheless they may result in temporary, long-term, or permanent gonadal toxicity in male patients^1–3^. Damage to spermatogenesis can result from either direct radiation of the testes or the scattered dose received during the radiation treatment of cancers, such as prostate, bladder, rectal, and bone cancers^4^. Furthermore, the male reproductive potential continues to be adversely affected not only by clinical, but also environmental and occupational radiation exposures^5,6^. From a radiobiological perspective, however, the underlying mechanisms of infertility following radiation exposure remain unclear^7^. The preservation of male fertility following exposure to various radiation types, particularly radiation treatment for cancer, has therefore been one of the most significant challenges in the field of basic, translational, and clinical radiation research.

Radiation-induced effects on biological tissues were recognised immediately after the pivotal discovery of X-rays by Wilhelm Röntgen in 1895^8^. In general, radiation-induced effects at the tissue level seem to be dose-dependent with or without a threshold^9–12^. However, there is a remarkable difference in tissue-level responses depending on whether radiation is delivered uniformly or non-uniformly. In 1909, Alban Köhler reported the clinical observations of the tissue-sparing response during “grid radiotherapy” in which spatially fractionated radiation is delivered using a grid-like pattern of beams^13,14^. Furthermore, since the establishment of the fundamental concept of “microbeam radiotherapy (MRT)” in the 1990s, which is based on a spatial fractionation of synchrotron-generated X-ray microbeams at the microscale level^15^, notable tissue-sparing effects (TSE) following exposure to micro-slit X-ray microbeams have been confirmed in a variety of species and tissue types^16–21^. Therefore, although the underlying mechanisms of TSE remain unclear, we hypothesize that TSE of MRT in the testes would be helpful for the preservation of male fertility, while delivering high doses of radiation to the tumor.

**Figure 1:**
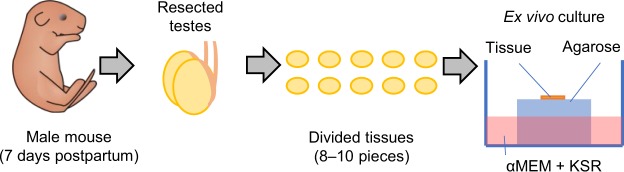
*Ex vivo* testes organ culture. Schematic representation of *ex vivo* testes organ culture. Testes were obtained from 7 days postpartum male mice. Resected testes were cut into 8–10 pieces, and then each piece was placed on an agarose gel block immersed in α-MEM medium containing KSR (KnockOut Serum Replacement).

To test our hypothesis, we show, for the first time, the TSE following MRT for preserving spermatogenesis and then elucidate the conditions required for eliciting them in the testes. For the purposes, we used a novel technical combination of synchrotron-generated X-ray microbeams and a unique *ex vivo* testes organ culture which enables to monitor the progress of spermatogenesis and to easily assess the radiation-induced impacts. In this study, we used a transgenic mouse model expressing acrosome-green fluorescent protein (Acr-GFP) which is a meiosis-specific biomarker^22,23^. We coupled this with a novel *ex vivo* testes organ culture technique, developed in 2011 to produce fully functional sperm *in vitro* (Fig. 1, and Supplementary Fig. 1, 2)^24^. This allows clear and easy monitoring of the process of spermatogenesis for more than one month^25^. As previously reported, this *ex vivo* model of spermatogenesis can reproduce the deterministic effects of radiation (*e.g*., temporary infertility and permanent sterility) following uniform exposure to conventional X-rays^26^.

**Figure 2:**
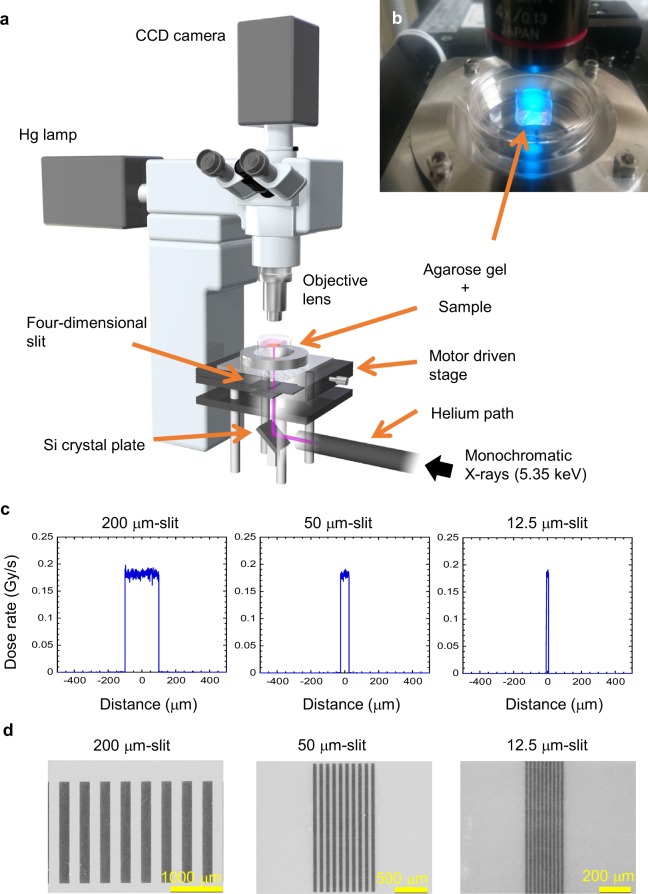
Synchrotron-generated X-ray microbeam irradiation settings. (**a**) Optical apparatus for X-ray microbeam irradiation, using the synchrotron beamline BL-27 at the Photon Factory, High Energy Accelerator Research Organization (KEK). (**b**) Photo of X-ray microbeam irradiation settings with *ex vivo* testes organ cultures. (**c**) Dose profiles of the 200, 50, and 12.5 μm-width microbeams, calculated with PHITS code. The beam intensity was essentially flat within the beam width. The deviation of the dose was about ± 6% of the averaged dose. Due to the very short range of secondary electrons (1.1 μm maximum) produced by the 5.35 keV X-ray exposure, the doses delivered outside of the irradiated area was negligible (<0.25%). (**d**) Dose profiles of the 200, 50, and 12.5 μm-width microbeams were also confirmed using Gafchromic XR-RV3 radiochromic film (Ashland Inc., Covington, KY, USA). Scale bars, 1000, 500, and 200 μm.

We also used a 5.35 keV monochromatic X-ray microbeam irradiator at the Photon Factory synchrotron facility based at the High Energy Accelerator Research Organisation (Tsukuba, Japan) (Fig. 2a,b)^27^. In the present study, we confirmed that the radiation-induced biological effects on spermatogenesis (*e.g*., temporary infertility and permanent sterility) in *ex vivo* replicant samples following uniform exposure to the synchrotron X-ray beams are dose-dependent (see Supplementary Figs 3, 4).

In 1995 Slatkin and his colleagues of the Brookhaven National Laboratory (Upton, NY, USA) first reported that those brain cells showed unusual resistance to necrosis after exposure to synchrotron-generated micro-slit X-ray microbeams with an effective energy region from 32 to 126 keV, a critical energy of 48.5 keV^16^. These high energy X-rays interact with matter through both photoelectric process and Compton scattering equally^28^. The Compton scattering can produce scattering photons and recoil electrons in a similar energy region of incident X-rays^29^. This indicates that, even when irradiated with micro-slit X-ray microbeams, the non-irradiated part (valley part) in the tissue also received a certain dose though the secondary ejected particles. However, our experimental procedure using lower energy (5.35 keV) X-rays in the Photon Factory made it possible to separately investigate the responses of the irradiated and the non-irradiated areas in the tissue^26^.

As shown in Fig. 2c, to investigate biological responses in non-uniform radiation fields, we performed high-precision 200, 50 and 12.5 μm-slit irradiation, where approximately 50% of the sample was irradiated via a four-dimensional slit system of monochromatic X-ray microbeam irradiator. The dose profiles of these microbeams were calculated with a Monte Carlo particle transport simulation code, PHITS ver. 2.96^30^. The dose profiles were also confirmed using radiochromic film (Fig. 2d). For MRT, the adequacy of the procedure of averaging the non-homogeneous peak and valley doses^31^, or the adequacy of using the valley dose as a value that is biologically equivalent to that of a seamless, broad beam exposure of live mammalian cells or organisms has been postulated, but not has not been formally and unequivocally confirmed by preclinical experiments.

## Results

### Live-tissue imaging reveals the tissue-sparing effects for spermatogenesis

**Figure 3:**
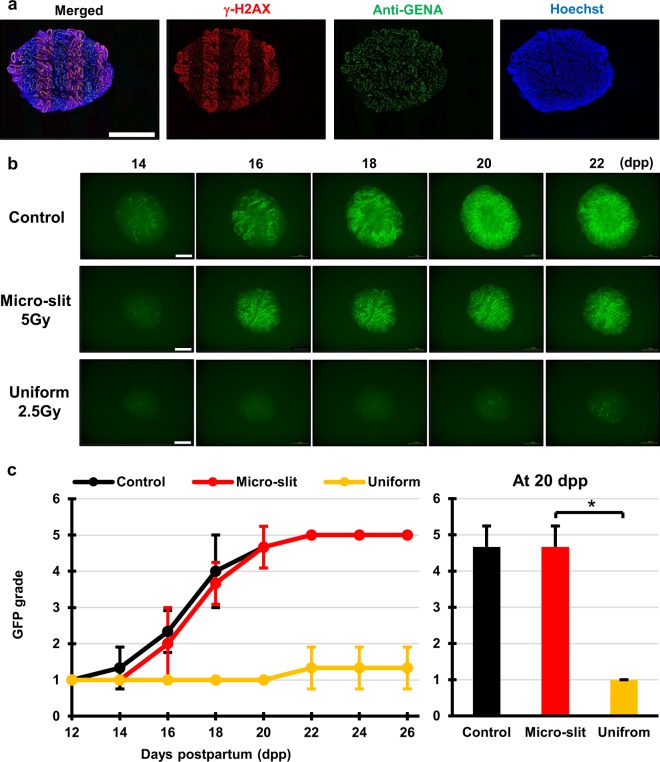
Tissue-sparing effect of micro-slit radiation in spermatogenesis. (**a**) Immunohistochemical images of *ex vivo* testes culture tissues 1 h after 10 Gy 200 μm-slit X-ray microbeam irradiation. Staining for γ-H2AX, Anti-GENA (anti-germ cell-specific antigen antibody), and Hoechst (for DNA) is shown as red, green, and blue, respectively. Scale bars, 500 μm. (**b**) Representative images show Acr-GFP expression changes in single cultures following 0 Gy (control), 5 Gy micro-slit (50%), and 2.5 Gy uniform (100%) X-ray irradiation, from 14 to 22 days postpartum (dpp). Scale bars, 500 μm. (**c**) Chronological changes in Acr-GFP expression after the micro-slit and the uniform X-ray irradiation. A minimum of three tissue samples each from different donor mice were used for each experiment. Data represent the mean GFP expression ± SD. Asterisk indicates P < 0.01.

First, using the micro-slit X-ray microbeams and the *ex vivo* testes organ culture, we tested the TSE following MRT for preserving spermatogenesis. Testes samples were obtained from around 7 days postpartum (dpp) *Acr-GFP* transgenic male mice, and each sample was cut into 8–10 tissue pieces approximately 1 mm3 in size for *ex vivo* organ culture^25^. To observe radiation-induced effects on spermatogenesis, the cultured samples were irradiated at 8 dpp. The staining of γ-H2AX was used to confirm the 50% 200 μm-slit irradiated areas in the cultured tissue. As shown in Fig. 3a, the distribution of immune-stained γ-H2AX in the sample was a good approximation of the shape of the MRT irradiation patterns.

Our live-tissue imaging revealed that the dose of 2.5 Gy applied in uniform mode almost completely obliterated the fluorescence signal of Acr-GFP from 14 to 22 dpp, whereas in the micro-slit irradiated from 16 to 22 dpp, the fluorescence signal remained similar to that of non-irradiated controls, indicating the occurrence of a significant TSE for spermatogenesis (Fig. 3c). To our knowledge, this is the first study to visualize the TSE for spermatogenesis in response to non-uniform radiation fields such as MRT.

### Non-irradiated spermatogonial cells are required for the effective tissue-sparing effects for spermatogenesis

**Figure 4:**
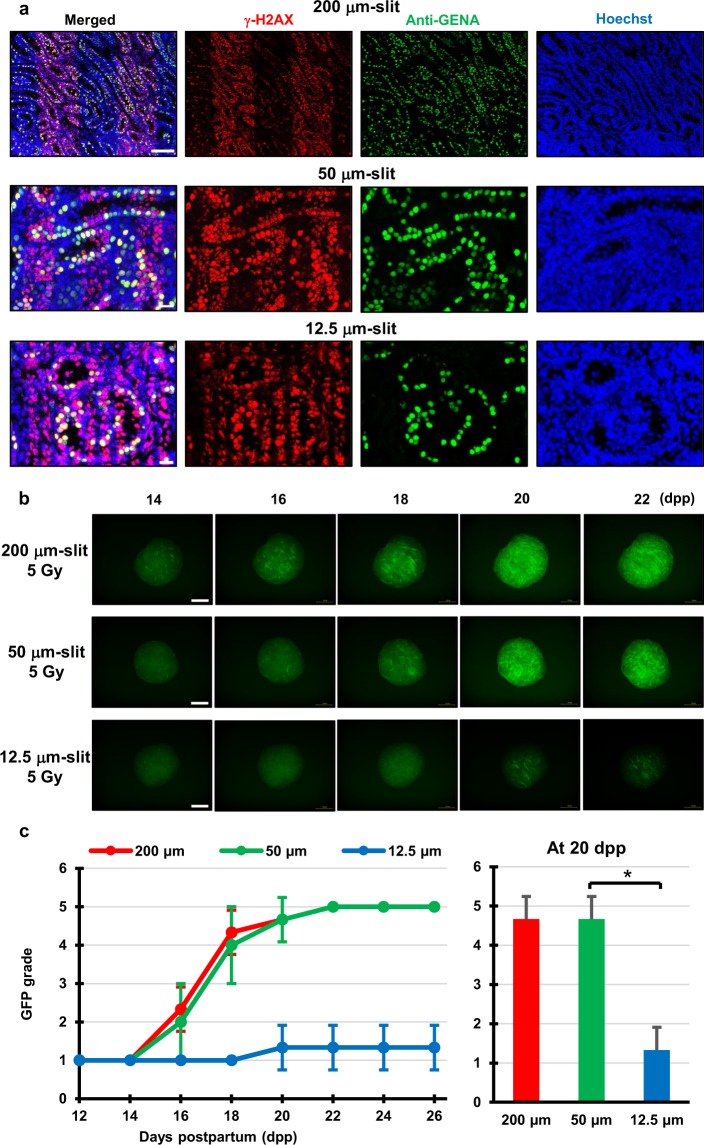
Difference of tissue-sparing effect between 200, 50, and 12.5 μm-slit radiation. (**a**) Immunohistochemical images of *ex vivo* testes culture tissues 1 h after 10 Gy 200, 50 and 12.5 μm-slit X-ray microbeam irradiation. Staining for γ-H2AX, Anti-GENA (anti-germ cell-specific antigen antibody), and Hoechst (for DNA) is shown as red, green, and blue, respectively. Scale bars, 100 μm (upper); 20 μm (middle and lower). (**b**) Representative images showing Acr-GFP expression in single cultures following 0 Gy (control), 5 Gy 200, 50 and 12.5 μm-slit (50%), and 2.5 Gy uniform X-ray irradiation (100%) obtained at 14, 16, 18, 20 and 22 dpp. Scale bars, 500 μm. (**c**) Chronological Acr-GFP expression changes after the 5 Gy 200, 50 and 12.5 μm-slit (50%) X-ray irradiation. A minimum of three tissue samples each from different donor mice were used for each experiment. Data represent the mean Acr-GFP expression ± SD. Asterisk indicates P < 0.01.

Next, we elucidated the conditions required to elicit the effective TSE following MRT by changing the micro-slit width from 200 to 12.5 μm. The staining of γ-H2AX was used to confirm the 50% 200, 50, and 12.5 μm-slit irradiated areas in the cultured sample. As shown in Fig. 4a, the distribution of immune-stained γ-H2AX in the sample was a good approximation of the shape of the MRT irradiation patterns.

Our live-tissue imaging reveals that the Acr-GFP expression changes significantly differed for the 12.5 μm-slit irradiated tissue when compared with either the 200 or the 50 μm-slit irradiated tissues (Fig. 4b,c). Because the average diameter of spermatogonial cells at 7–8 dpp is approximately 15 μm^32^, some of the spermatogonial cells were unirradiated in the 200 and 50 μm irradiated tissues, whereas almost all the spermatogonial cells were partially or completely irradiated in the 12.5 μm-slit irradiated tissues (Fig. 4a). Thus, the result showed that the effective TSE for spermatogenesis required a non-irradiated spermatogonial cell population.

Periodic Acid-Schiff (PSA) staining, commonly used to visualize the changes of the developing acrosome during acrosome-genesis^33^, can reveal the presence of round or elongating spermatids. As shown in Supplementary Fig. 5, we confirmed the presence of round or elongating spermatids in the culture tissues following exposure to 200 and 50 μm-slit radiation and incubation for around 35 days which is enough long for the period of spermatogenesis. Offspring can be produced by sperm cells or round or elongating spermatids in *ex vivo* cultured tissues via intracytoplasmic sperm injection (ICSI) or round spermatid injection (ROSI) techniques^24^. Therefore, the differentiated spermatogonial cells produced in the micro-slit irradiated tissue due to the tissue-sparing effects could produce offspring, showing the preservation of male fertility.

### Spermatogonial cell migration for maintaining spermatogenesis in the 50% irradiated tissues

**Figure 5:**
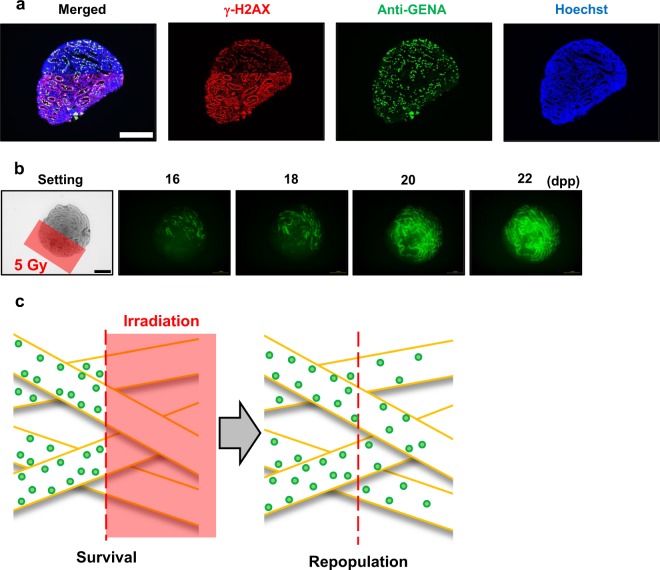
Chronological GFP expression changes after X-ray half-irradiation. (**a**) Immunohistochemical images of *ex vivo* testis tissues 1 h after 10 Gy X-ray 50% irradiation using X-ray microbeams. Staining for γ-H2AX, Anti-GENA and Hoechst is shown as red, green and blue, respectively. Scale bars, 500 μm. (**b**) Representative images showing Acr-GFP expression in single cultures of testes exposed to 5 Gy 50% irradiated X-ray microbeams obtained at 16, 18, 20 and 22 dpp. In the irradiation setting image, the irradiated area is shown as a red square. The Acr-GFP expression areas expanded from the non-irradiated to the irradiated areas due to the tissue-sparing effect. Scale bars, 500 μm. (**c**) Schematic representation of the two-step process of the tissue-sparing effect in spermatogenesis. First, spermatogonial cells survive in the non-irradiated areas, whereas they are eliminated in the irradiated areas after irradiation. The surviving cells then migrate to the irradiated area via the seminiferous tubules. Thus, the spermatogonial cells require these two steps, survival and migration, for an effective tissue-sparing effect in spermatogenesis.

To investigate the TSE expanding process in the progress of spermatogenesis, we imaged the TSE for spermatogenesis after 50% irradiation of a tissue culture expressing Acr-GFP (*i.e*., irradiation of one of the two dimidiate parts). The staining of γ-H2AX was used to confirm the 50% irradiated areas in the cultured sample. As shown in Fig. 5a, the distribution of immune-stained γ-H2AX in the sample was a good approximation of the shape of the irradiation patterns.

Our live-tissue imaging reveals that after 50% irradiation of the cultured tissue, the areas expressing Acr-GFP were visibly expanded from the non-irradiated to the irradiated areas (Fig. 5b). The γ-H2AX staining also revealed the possibility of spermatogonial cell migration from the non-irradiated to the irradiated areas 18 h after 50% irradiation (Supplementary Fig. 6). As shown in Fig. 5c, this result indicated that the effective TSE expansion required the migration of the non-irradiated stem cells via the seminiferous tubules.

## Discussion

Our high-precision microbeam analysis demonstrated that the dynamics of the non-irradiated germ cell population via seminiferous tubules would be essential for the effective testes TSE following MRT for preserving spermatogenesis. Previous studies of seminiferous tubule repopulation following irradiation indicated that spermatogonial stem cells begin colony formation soon after the destruction of differentiated germ cells^34^. Furthermore, male mouse germ cell transplantation was first performed by Ralph Brinster and his colleagues in 1994 and resulted in donor cell spermatogenesis in the recipient testes, showing that the dynamics of spermatogonial stem cell (SSC) via seminiferous tubules are important for maintaining spermatogenesis^35^. According to the series of SSC transplantation studies, the colonization process of stem cells can be divided into three continuous phases^36^; (i) During the initial week, transplanted cells were randomly distributed throughout the tubules, and a small number reached the basement membrane, (ii) From 1 week to 1 month, donor cells on the basement membrane divided and formed a monolayer network, and (iii) Beginning at about 1 month and continuing throughout the observation period, cells in the center of the network differentiated extensively and established a colony of spermatogenesis, which expanded laterally by repeating phase two and then three. These studies supported our findings that the TSE following exposure to radiation requires the two steps: survival of spermatogonial cells in the non-irradiated areas and migration of these cells via the seminiferous tubules.

**Figure 6:**
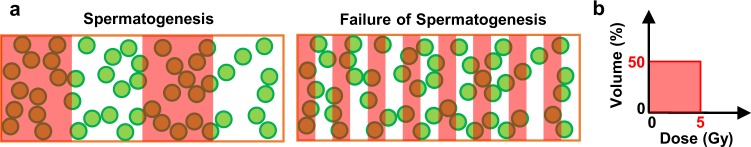
Failure of conventional radiological does-volume concept in the *ex vivo* spermatogenesis. (**a**) Two different 50% micro-slit irradiation patterns of the *ex vivo* testes tissues. They had the same irradiated area of 50% and the same total irradiation dose, of 2.5 Gy at the whole tissue level. Green circles and red squares are germ cells and irradiated areas, respectively. (**b**) Dose-volume histograms of the two 50% micro-slit irradiation patterns. These two irradiation scenarios cannot be distinguished by their histograms, showing the logical failure of conventional radiation dose-volume concept at the microscale level.

Our technical combination of a unique *ex vivo* testes organ culture and high-precision X-ray microbeams will provide novel insights of radiation-induced deterministic and stochastic effects on male reproduction, as well as of the transgenerational effects on spermatogonial stem cells. As shown in Fig. 6a, we noticed that there are theoretically two different patterns at the microscale level for the cultured testes tissues in which there are spermatogonial stem cells after 50% 5 Gy irradiation. These cells can migrate and proliferate in the tissue, and regulate the TSE following for spermatogenesis exposure to spatially fractionated radiation. On the one hand, in the left pattern, some of the spermatogonial cells survive after irradiation, and an effective TSE occurs for spermatogenesis. On the other hand, in the right pattern, all cells are partially or completely irradiated, and according to our experimental data, no tissue-sparing response is observed. However, according to the conventional radiation dose-volume metric and biological models, these two scenarios cannot be distinguished, because their radiation dose-volume histograms, which are plots of cumulative dose-volume frequency distributions commonly used in radiotherapy planning^37^, are identical (*i.e*., they had the same irradiated volume ratio, 50%, and received the same total dose, 2.5 Gy at the whole tissue level) (Fig. 6b). Thus, our findings showed a counterexample, suggesting the logical failure of the conventional radiation dose-volume concept at the microscale level.

In conclusion, we showed, for the first time, the TSE in the *ex vivo* testes model following MRT for male fertility preservation. The survival and potentially migration of the non-irradiated germ cells in the irradiated testes are required for the TSE for spermatogenesis, indicating the clinical potential of spatially-fractionated radiotherapeutic techniques, including MRT, for preserving male fertility in cancer treatment. The technical combination of high-precision microbeam analysis and specific *ex vivo* organ models will be of use in further investigation of the radiation-induced acute and late effects on specific physiological functions.

## Methods

### Animal model

The Acr-GFP transgenic mice were obtained from RIKEN BRC, Tsukuba, Japan, through the National BioResource Project of MEXT, Japan. These mice express the marker GFP specific for meiosis, which is useful for monitoring the progress of spermatogenesis. All animal experiments conformed to the Guide for the Care and Use of Laboratory Animals and were approved by the Institutional Committee of Laboratory Animal Experimentation.

### ***Ex vivo*** testes organ culture

Testes samples were obtained from 7 days postpartum (dpp) *Acr-GFP* transgenic mice, and each sample was cut into 8–10 tissue pieces approximately 1 mm3 in size^25^. Each tissue piece was immediately placed on a 1.5% agarose gel block immersed in 0.5 mL of α-MEM medium containing 10% KSR (KnockOut Serum Replacement, Thermo Fisher Scientific K.K, Yokohama, Japan), 1% antibiotic mixture solution (Antibiotic-Antimycotic, Thermo Fisher Scientific K.K.), and 0.2 mM NaHCO_3_ in a 12-well culture dish^25^. Samples were cultured in a humidified incubator at 34 °C under an atmosphere of 95% air and 5% CO_2_^25^. This unique *ex vivo* testes organ culture method, developed in 2011 to produce fully functional sperm *in vitro*^24^, allows clear and easy microscopic monitoring of the process of spermatogenesis for more than 40 days.

As shown in Supplementary Fig. 1, the size and shape of cultured sample before irradiation were examined with a 3D laser scanning microscope (VK-X250, KEYENCE, Osaka, Japan) (n = 1). The sample with a disk-like shape were approximately 200 μm thickness. The diameter was around 2000–2500 μm.

### Live-tissue imaging

The cultured samples were observed by fluorescence microscopy (BZ-X700, KEYENCE) with an x4 magnification objective lens to capture GFP-fluorescent and bright-field images. After the observation at different time points, samples were returned to the incubator and images acquired on alternate days. The exposure times are 1.5 s for the GFP-fluorescent images and 150 ms for the bright-field images.

The effects of the phototoxicity of GFP excitation light from fluorescence microscopy on live tissues should be considered. With PAS staining, we investigated how the phototoxicity of GFP excitation light affected to the observed area. The GFP-fluorescent and bright-field images acquired on alternate days showed almost intact spermatogenesis in the cultured samples, indicating that the phototoxicity was negligible.

PAS staining is commonly used to visualize the changes of the developing acrosome during acrosome-genesis and can reveal the presence of round or elongating spermatids. Tissues were fixed with Bouin’s fixative (Muto Pure Chemicals, Tokyo, Japan) and embedded in paraffin. One section showing the largest cut surface was selected for each specimen and stained with PAS-hematoxylin.

### Synchrotron-generated X-ray beams

To investigate the TSE following MRT for preserving spermatogenesis, we used a 5.35 keV monochromatic X-ray microbeam irradiator at the Photon Factory synchrotron facility of the High Energy Accelerator Research Organisation (KEK) in Japan (Fig. 2a,b)^27^. The microbeam work described in this paper was approved by the Photon Factory Program Advisory Committee (Proposal No. 2017P001).

The 5.35 keV monochromatic X-rays were guided in vertical direction through a helium path pipe and reflected to upper direction by Bragg diffraction of a single Si crystal plane (311). The sample dish was set on a pulse motor driven stage. The beam width was variable using a remote-controlled four-dimensional slit. The exposure (photon fluence) was determined by measuring the X-ray beam intensity upstream of the slit system using a specially designed air-free ionization with parallel-plate-collecting-electrodes 30 mm length with a 40 mm space between the electrodes^27^. A silicon photodiode (AXUV-100, International Radiation Detectors, Torrance, CA, USA) was used as a second detector for the intensity measurement at both the incident beam position (upstream of the slit) and microscope stage (downstream of the slit). The photodiode current at the sample position was then converted to exposure. The value was approximately 7.7 × 10^−3^ C/kg/s, confirmed by photon flux (9.3 × 10^3^ photons/s/100 μm^2^). The exposure applied to the sample was 0.26 C/kg, which roughly corresponds to an absorbed dose of 5 Gy. The composition of soft tissue given by the International Commission on Radiation Units and Measurements (ICRU)^38^ was used for the dose calculation. Based on the beam attenuation along depth direction of the sample, the dose at the bottom (approximately 200 μm from the surface) was about 53% of that at the sample surface.

### Micro-slit irradiation settings

To perform high-precision X-ray microbeam analysis, as shown in Fig. 2c, we performed high-precision 200, 50 and 12.5 μm-slit irradiation, where approximately 50% of the sample was irradiated (using center-to-center distances of 400, 100 and 25 μm, respectively). The dose profiles were calculated with a Monte Carlo particle transport simulation code, PHITS ver. 2.96^30^ and also confirmed using Gafchromic XR-RV3 radiochromic film (Ashland Inc., Covington, KY, USA).

To observe the TSE of MRT for preserving spermatogenesis, as shown in Fig. 2, the cultured samples were irradiated at 8 dpp with uniform exposure to 2.5 Gy (n = 3) and 50% micro-slit exposure to 5 Gy, namely 2.5 Gy at the whole tissue level, (n = 4) or left nonirradiated (n = 3). Furthermore, to further investigate the TSE for spermatogenesis, the cultured samples were irradiated with 50% 200 (n = 3), 50 (n = 4), and 12.5 μm-slit exposure to 5 Gy (n = 4). A single sample from each mouse testes was randomly assigned to an experimental group. A minimum of 3 tissue samples each from different donor mice were used for each experiment, however, contaminated samples were excluded.

### Evaluation of GFP expression

The Acr-GFP expressions in the culture tissue show the progression of spermatogenesis, and in the unirradiated cultures, the peak expression occurred at around 18–22 dpp. The observation of GFP expression along the tubules was designated as a sign of spermatogenesis. The expression was classified into 6 grades, 0–5, based on the expression area: 0, –20, –40, –60, –80, and –100%, respectively^39^. The central area was omitted from the evaluation because this area in many cases lacked GFP expression due to spatial and nutrient flow restrictions (see Supplementary Fig. 2)^24^. It is one of the technical limitations of the *ex vivo* testes organ culture method for monitoring the process of spermatogenesis.

### Statistical analysis

One-way analysis of variance (ANOVA) was used to assess differences in the GFP expression grade. Values with P < 0.05 were considered to indicate a significant difference.

### Immuno-histochemical analysis

For immunofluorescence staining, cultured samples (from three donor mice) fixed with 4% (w/v%) paraformaldehyde in phosphate-buffered saline (PBS) at 4 °C for 6 h or overnight were cryo-embedded in optimal cutting temperature (OCT) compound (Sakura Finetek, Tokyo, Japan) and cut into 7 μm-thick sections. Sections were washed once with PBS for 5 min and in 0.2% PBT four times for 10 min each. Then, sections were blocked with Image-iT FX Signal Enhancer (Thermo Fisher Scientific, Waltham, MA, USA) for 30 min and incubated overnight at 4 °C with the following first antibodies: Rabbit anti-γ-H2AX antibody (1:1000; Abcam, Cambridge, UK), Rat anti-GENA antibody, mouse clone TRA98 (1:1000; BioAcademia, Osaka, Japan), Rabbit anti-MVH (1:400; Abcam), Goat anti-GFRα1 antibody (1:200; R&D Systems, Minneapolis, MN, USA), Chicken anti-GFP antibody (1:1000; Abcam), and Rat anti-GFP antibody (1:1000; Nacalai tesque, Kyoto, Japan). The anti-γ-H2AX was used to detect DNA double strand breaks (DSBs) in the nucleus^40^. The anti-GENA and anti-MVH antibodies were used to detect germ cells^41,42^. The anti-GFRα1 antibodies were also used to detect spermatogonial stem cells (SSCs)^43,44^. Nuclei were counterstained with Hoechst 33342 (1 μg/ml; bisBenzimide H 33342)^45^.

After being rinsed four times with PBS containing Triton X-100 (0.2%), the following secondary antibodies were used; goat anti-rabbit IgG, goat anti-rat IgG, and donkey anti-goat IgG, conjugated to Alexa Fluor 488, Alexa Fluor 555, or Alexa Fluor 647 (1:200; Thermo Fisher Scientific). The immunologically stained samples were mounted on slides in ProLong Diamond Antifade Mountant (Thermo Fisher Scientific).

### TUNEL assay

Using TUNEL labelling, apoptotic cells were detected with the *In Situ* Cell Death Detection Kit, TMR red (Roche, Indianapolis, IN, USA)^46^. After this apoptosis labelling, immunofluorescence staining was performed.

## Supplementary information

Supplementary Information
