# Supplementary material for: High-precision microbeam radiotherapy reveals testicular tissue-sparing effects for male fertility preservation
Source: Sci Rep. 2019 Oct 1;9:12618. doi: 10.1038/s41598-019-48772-3 (PMC6773706; doi:10.1038/s41598-019-48772-3)
Supplement: Supplementary file 1 — Supplementary Information [file 41598_2019_48772_MOESM1_ESM.docx]

**High-precision microbeam radiotherapy reveals testicular tissue-sparing effects for male fertility preservation**

Hisanori Fukunaga, Kiichi Kaminaga, Takuya Sato, Karl T. Butterworth, Ritsuko Watanabe, Noriko Usami, Takehiko Ogawa, Akinari Yokoya & Kevin M. Prise

^*^ Correspondence and requests for materials should be addressed to A.Y. (email: yokoya.akinari@qst.go.jp) or to K.M.P. (email: k.prise@qub.ac.uk).

**Supplementary Materials:**

Supplementary Figure 1–6

**
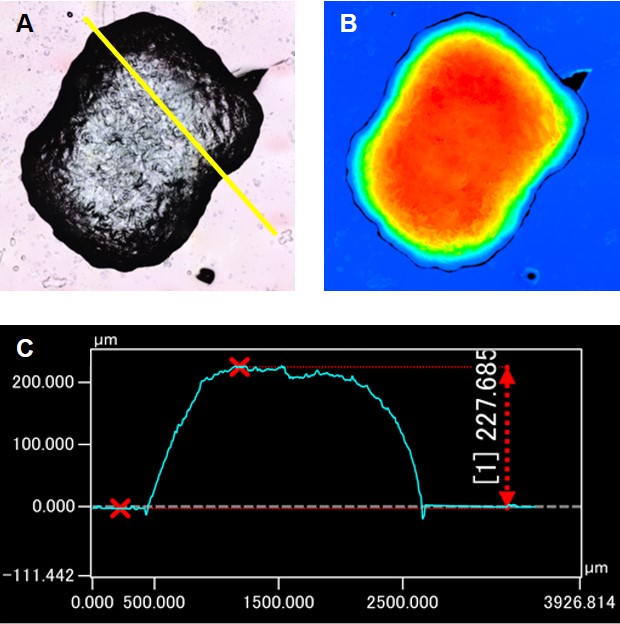
**

**Supplementary Figure 1****.** **Shape of testes tissue in the *ex vivo* organ culture.**

**(A, B)** Laser microscopic images of testis tissue. Measured line to detect the thickness is shown in yellow.

**(C)** Thickness of tissue on the measured line. The tissue has approximately 200 μm thickness and a disk-like shape. The diameter is around 2000–2500 μm.


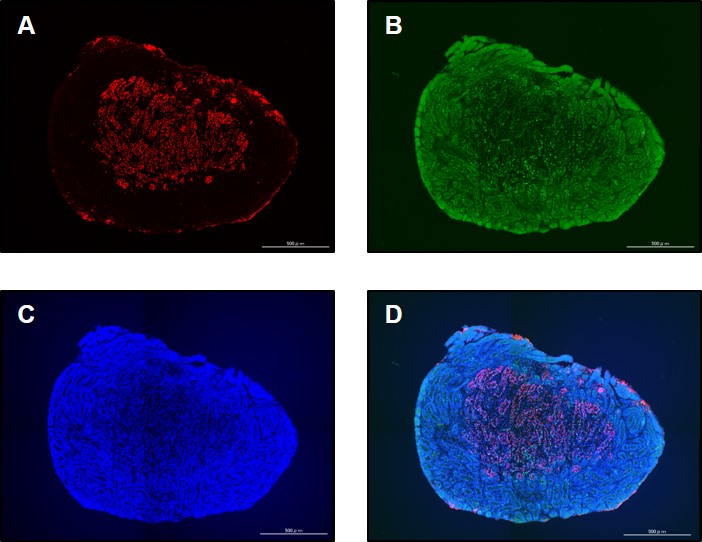


**Supplementary Figure 2.** **Technical limitations of the *ex vivo* testes organ culture.**

(**A**) TUNEL, (**B**) Anti-GENA, (**C**) Hoechst, and (**D**) merged staining images 24 h after starting the culture. The centers and edges of tissues showed tissue damage and apoptosis due to the technical limitations of the *ex vivo* testes organ culture method. Scale bar; 500 μm.


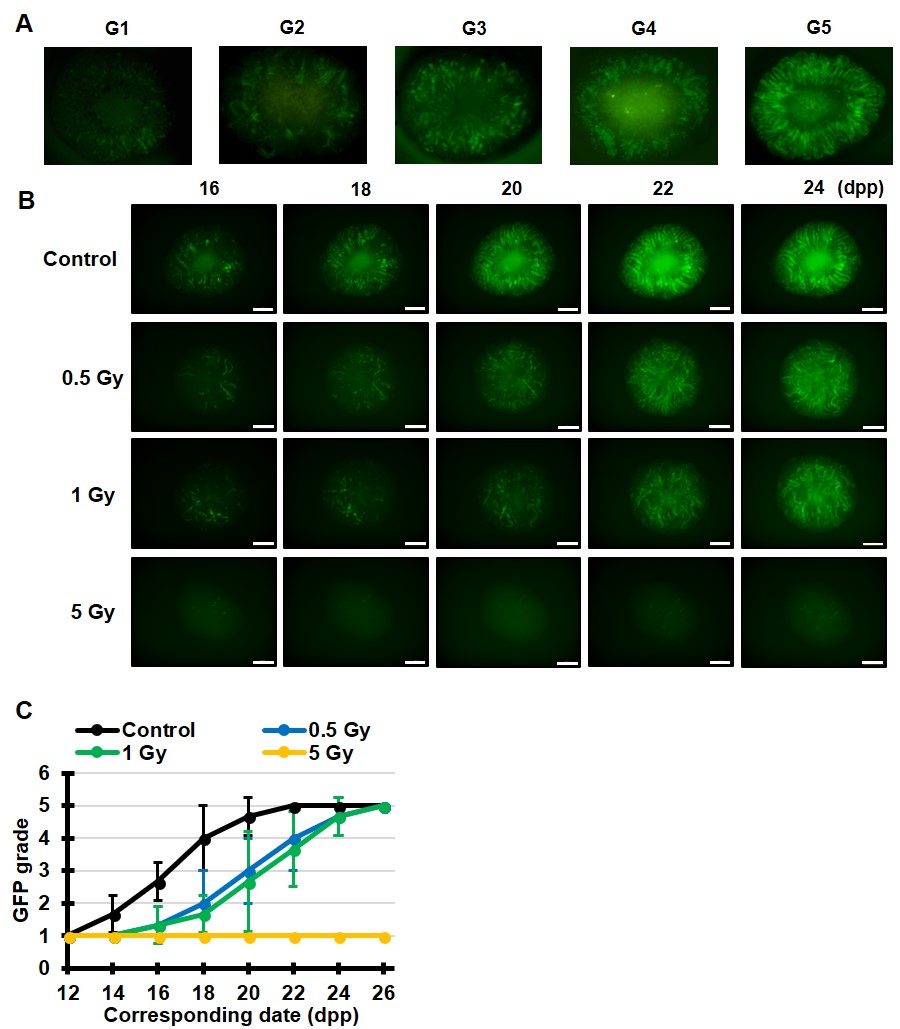


**Supplementary Figure 3.** **Chronological GFP expression changes after uniform irradiation using synchrotron-generate X-ray microbeam irradiator.**

(**A**) GFP expression grade. The observation of GFP expression along the tubules was designated as a sign of spermatogenesis. The expression was classified into 6 grades, 0–5, based on the expression area: 0–10, 11–30, 31–50, 51–70, and 71–100%, respectively respectively. The central area was omitted from the evaluation because this area in many cases lacked GFP expression due to spatial and nutrient flow restrictions. It is one of the technical limitations of the *ex vivo* testes organ culture method for monitoring the process of spermatogenesis.

(**B**) Representative images show Acr-GFP expression changes in single cultures of testis after 0 (control), 0.5, 1, and 5 Gy uniform X-ray irradiation, from 16 to 24 dpp. Scale bars, 500 μm.

(**C**) Chronological changes in Acr-GFP expression. A delay in the dose-dependent peak expression was confirmed by observation after irradiation at dose of 0.5 Gy or greater. Also, the dose-dependent tissue damage and decrease in the area of GFP expression were also confirmed after 5 Gy irradiation. These demonstrate reversible and irreversible radiobiological effects which may represent the clinical conditions of temporary infertility and permanent sterility. A minimum of three tissue samples each from different donor mouse were used for each experiment. Data represent the mean GFP expression ± SD.


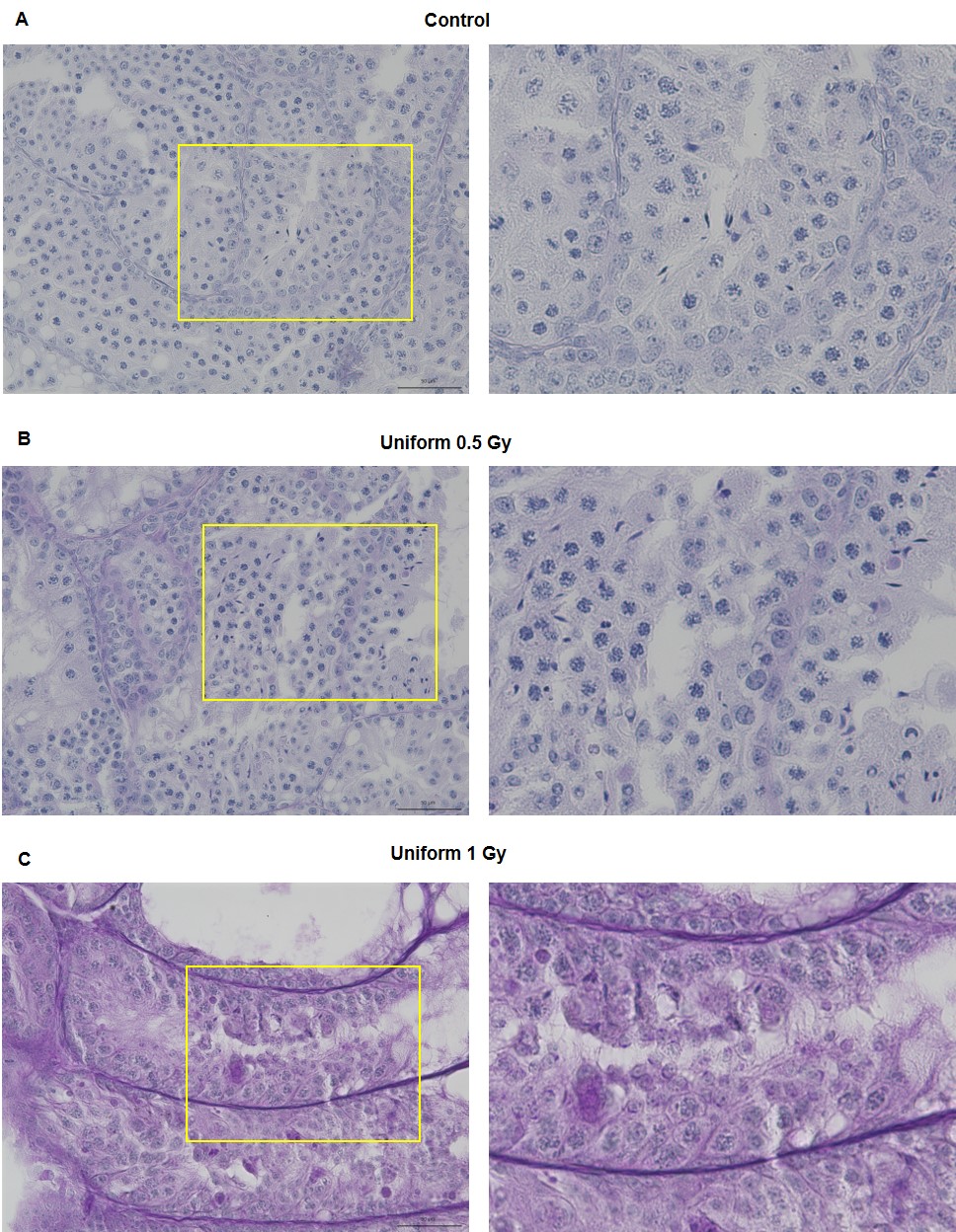


**Supplementary Figure 4.** **PAS staining images of the *ex vivo* culture tissues after uniform X-ray irradiation.**

(**A-C**) Round and elongating spermatids were detected in cultures after 0 (control) (**A**), 0.5 (**B**), and 1 Gy uniform irradiation (yellow squares) (**C**). The yellow box in the left images is enlarged in the right images, showing round or elongating spermatids (white arrows). Scale bars, 50 μm.


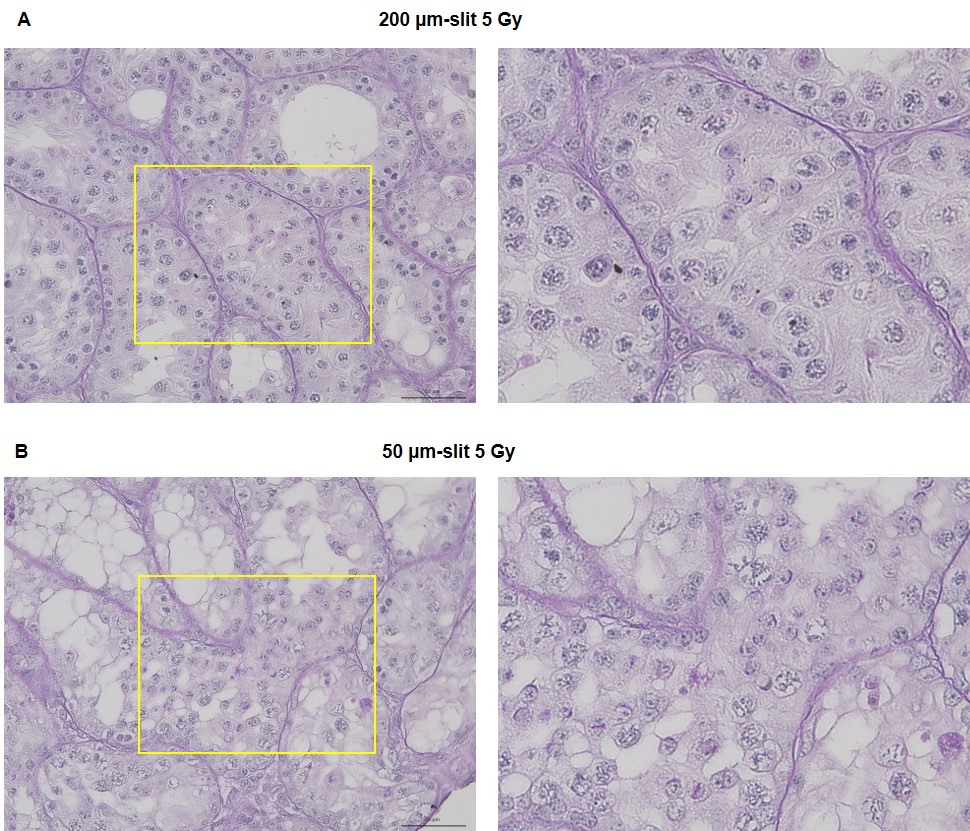


**Supplementary Figure 5.** **PAS staining images of the *ex vivo* culture tissues after 200 and 50 μm-slit 5 Gy irradiation and spermatogenesis.**

Round and elongating spermatids in the culture following 200 and 50 μm-slit 5 Gy irradiation and incubation for more than 30 days. The yellow box in the left images is enlarged in the right images, showing round or elongating spermatids (white arrows). Scale bars, 50 μm.


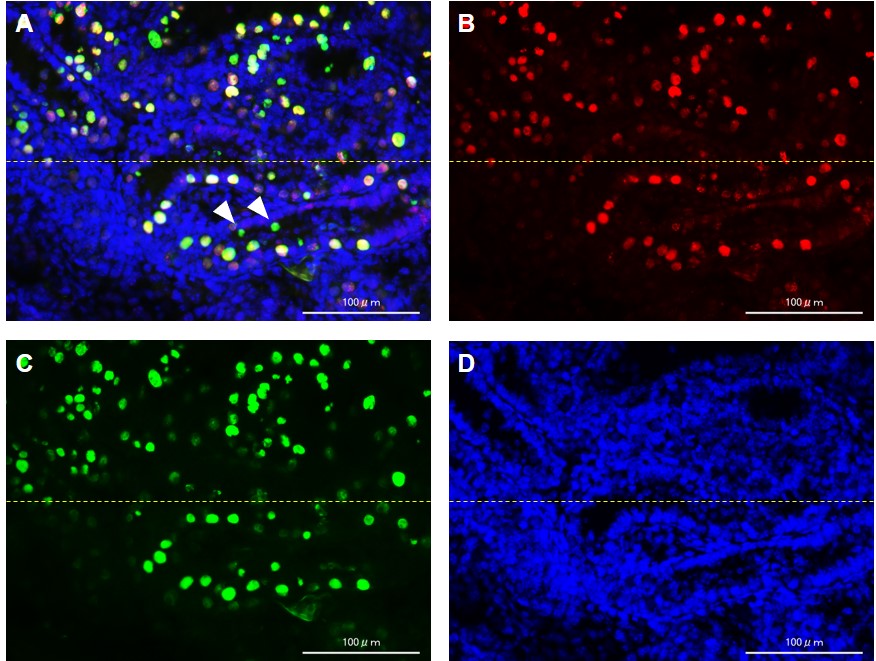


**Supplementary Figure 6.** **Immunohistochemical images of the *ex vivo* culture tissues 18 h after 10 Gy X-ray half-irradiation.**

(**A-D**) Immunohistochemical images of *ex vivo* testis tissues 18 h after 10 Gy X-ray 50% irradiation using X-ray microbeams. Staining for γ-H2AX (**B**), Anti-GENA (**C**) and Hoechst (**D**) is shown as red, green and blue, respectively. Yellow dot lines are the borders between the non-irradiated (upper) and irradiated areas (lower) after half-irradiation. DNA repair in spermatogonial cells (Tra98-positive cells) is not functional, thus the γ-H2AX-negative spermatogonial cells (white arrows) in the irradiated area indicate their migration from the non-irradiated area after half-irradiation. Scale bars, 100 μm.
